# Differential effect of pre-pregnancy low BMI on fetal macrosomia: a population-based cohort study

**DOI:** 10.1186/s12916-021-02046-w

**Published:** 2021-08-04

**Authors:** Guoju Li, Yuhan Xing, Guolan Wang, Jun Zhang, Qin Wu, Wei Ni, Na Jiao, Wenjing Chen, Qing Liu, Li Gao, Zhenhong Zhang, Yao Wang, Quansheng Xing

**Affiliations:** 1 Qingdao Women and Children’s Hospital, Qingdao University, No.6 Tongfu Road, Qingdao, 266000 Shandong Province China; 2 Department of Paediatrics, Faculty of Medicine, The Chinese University of Hong Kong, Hong Kong, China; 3 Qingdao Women and Children’s Health Care and Family Planning Service Center, Qingdao, Shandong Province China; 4 Public Health School, Medical College of Qingdao University, Qingdao, China

**Keywords:** Macrosomia, Pre-pregnancy BMI, Gestational diabetes mellitus, Parity, Maternal age

## Abstract

**Background:**

The differential effect of pre-pregnancy low BMI on macrosomia has not been fully addressed. Herein, we conducted a city-wide population-based cohort study to illuminate the association between pre-pregnancy low BMI and macrosomia, stratifying by maternal age, parity, and GDM status.

**Methods:**

All pregnant women who paid their first prenatal visit to the hospital in Qingdao during August 1, 2018, to June 30, 2020, were recruited to this study. The interactive effect of maternal age and pre-pregnancy low BMI on macrosomia was evaluated using logistic regression models, followed by strata-specific analyses.

**Results:**

A total of 105,768 mother-child pairs were included, and the proportion of fetal macrosomia was 11.66%. The interactive effect of maternal pre-pregnancy BMI and age was statistically significant on macrosomia irrespective of parity (nullipara: *P*_*adjusted*_=0.0265; multipara: *P*_*adjusted*_=0.0356). The protective effect of low BMI on macrosomia was most prominent among nullipara aged 35 years and above (aOR=0.16, 95% CI 0.05–0.49) and multipara aged 25 years and below (aOR=0.17, 95% CI 0.05–0.55). In nullipara without GDM, the risk estimates gradually declined with increasing conception age (20-to-24 years: aOR=0.64, 95% CI 0.51–0.80; 25-to-29 years: aOR=0.43 95% CI 0.36–0.52; 30-to-34 years: aOR=0.40 95% CI 0.29–0.53; and ≥35 years: aOR=0.19, 95% CI 0.06–0.60). A similar pattern could also be observed in nullipara with GDM, where the aOR for low BMI on macrosomia decreased from 0.54 (95% CI 0.32–0.93) in pregnant women aged 25–29 years to 0.30 (95% CI 0.12–0.75) among those aged 30–34 years. However, younger multiparous mothers, especially those aged 25 years and below without GDM (aOR=0.21, 95% CI 0.06–0.68), were more benefited from a lower BMI against the development of macrosomia.

**Conclusions:**

Maternal low BMI is inversely associated with macrosomia irrespective of maternal age and parity. The impact of pre-pregnancy low BMI on macrosomia varied by maternal age and parity. The protective effect of a lower maternal BMI against fetal macrosomia was more prominent in nulliparous mothers aged 35 years and above, whereas multiparous mothers younger than 25 years of age were more benefited.

## Background

Fetal macrosomia constitutes one of the leading causes of various infantile complications including fracture, perinatal asphyxia, cerebral hemorrhage, brachial plexus injury, and even death and is associated with long-term adverse outcomes such as obesity in children [1–6]. Previous studies have shown that mothers who gave birth to macrosomic infants had a greater risk of developing subsequent metabolic disorders such as diabetes and gestational diabetes mellitus (GDM) than mothers of infants with normal birth weight [7]. The incidence of macrosomia has been reported between 5 and 20% in developed countries, with an increase of 15–25% over the past few decades [8]. Particularly, an increasing trend of macrosomia prevalence has been documented in China, from 6.9% in 2007 to 7.8% in 2017 [9]. Given the immense societal and individual burden of macrosomia, identification of risk factors is of paramount importance for development of primary preventive strategies.

Risk factors contributing to fetal macrosomia include maternal overweight and obesity [10, 11], advanced conception age [12], GDM [13], and parity≥2 [14]. There has been mounting evidence on the association between pre-pregnancy overweight/obesity and macrosomia; nevertheless, whether pre-pregnancy low BMI also influence the risk of macrosomia is inconclusive [15, 16]. A previous study of over 7 million singleton live births suggested that the effect of pre-pregnant obesity on preterm birth differed by maternal age [17]. We therefore speculated that the impact of pre-pregnancy low BMI on macrosomia may also differ by conception age and other maternal factors. With the recent implementation of “two children policy” in China, it is conceivable that more women are going to have a second child at a more advanced age. Our previous study has shown that the proportion of multipara is 52.62% [18]. Therefore, the effects of pre-pregnancy low BMI, maternal age, and parity on the risk of macrosomia need to be investigated in a large dataset with substantial available information adjusting for potential confounding factors. The objective of this study is to determine the association between maternal pre-pregnancy low BMI and macrosomia, stratifying by maternal age, parity, and GDM status.

## Methods

### Study design and data sources

**Fig. 1:**
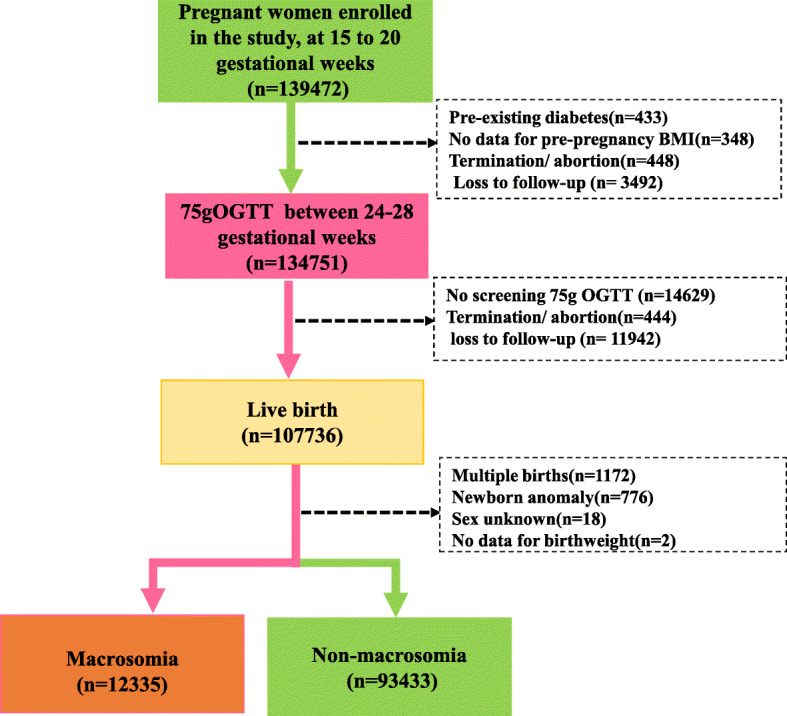
Study population and design

Pregnant women at 15 to 20 weeks of gestation (*n*=139,472) were recruited to this study during their first prenatal visit in Qingdao from August 1, 2018, to June 30, 2020. Data were registered in the Women and Children’s Health Care Center System, which was established in 2018 under the charge of Qingdao Women and Children’s Hospital. This registry provides comprehensive information covering results of regular health examinations, pre-pregnancy, and delivery details. Pregnant women and their detailed information at each gestation stage were identified and extracted using their ID number. Exclusion criteria included pregnant women with pre-existing diabetes (*n*=433), missing data for pre-pregnancy weight/height (*n*=348), termination/abortion before 24–28 gestational weeks (*n*=448), loss to follow-up before 24–28 gestational weeks (*n*=3492, no 75 g oral glucose tolerance test (OGTT) screening (*n*=14,629), termination/abortion (*n*=444), loss to follow=up (*n*=11,942), multiple births (*n*=1172), and dysmorphia (*n*=776) or missing key variables (*n*=20) (Fig. 1). A total of 105,768 singleton births were eligible for final analysis. Participation in the study was voluntary, and written informed consent was obtained from each study subject. This study was approved by the Institutional Review Board of Qingdao Women and Children’s Hospital Ethics (No. 002-2018-FEKY).

### Data collection

Detailed information of demographic characteristics, folic acid supplements, parity, alcohol drinking, tobacco smoking, and occupational physical activity were collected using standardized questionnaires. Meanwhile, responses from participants were checked by trained interviewers to improve the validity of the self-reported data. The primary outcome was macrosomia. Macrosomia was defined as an infant with birth weight ≥ 4000 g [19] and were further classified into three subgroups: grade 1: 4000–4499 g, grade 2: 4500–4999 g; grade 3: ≥ 5000 g [20]. Maternal pre-pregnancy BMI was calculated from self-reported values of height and weight before conception according to subjects’ answers to the questionnaire. Maternal pre-pregnancy BMI was categorized as low BMI (BMI<18.5 kg/m^2^), normal (BMI: 18.5–24.9 kg/m^2^), overweight (BMI: 25.0–29.9 kg/m^2^), and obese (BMI ≥30.0 kg/m^2^) based on the World Health Organization (WHO) criteria [21]. GDM was diagnosed in accordance with the International Association of Diabetes and Pregnancy Study Group recommendation (IADPSG) using 75 g 2-h OGTT: a fasting glucose ≥5.1 mmol/L, or a 1-h result ≥10.0 mmol/L, or a 2-h result ≥ 8.5 mmol/L [22]. Self-reported occupational physical activities were categorized into 3 levels: (1) light (mostly sitting for office work, e.g., secretary), (2) moderate (standing and walking, e.g., sale assistant, craftspeople), and (3) active (walking and lifting, heavy manual labor, e.g., industrial or farm worker) [23]. Maternal smoking was defined as mothers who smoked at least one cigarette per day and kept smoking for over 3 months before conception or kept smoking during gestation. Due to the very low prevalence rate of maternal smoking (before gestation 0.66% [*n*=693], during gestation 0.17% [*n*=176]), this factor was not included in the analysis. Paternal smoking was defined as fathers who smoked at least one cigarette per day and kept smoking for over 3 months. Maternal drinking was defined as mothers who drank alcohol regularly before or during gestation; paternal drinking was defined as fathers who drank alcohol regularly before conception.

### Statistical analysis

Logistic regression models were performed and odds ratios (OR, with 95% confidence intervals [CI]) were calculated to evaluate the risk associated with macrosomia. The interaction between maternal age and pre-pregnancy BMI on macrosomia was assessed using logistic regression models. If statistically significant interaction was found, strata-specific analysis would be further performed. Stratified analyses were performed based on parity and maternal age to determine disparities in relation to pre-pregnancy low BMI and macrosomia. In order to further clarify the association, the combined effect of GDM status and parity was evaluated in different age groups. *P* values of two-sided tests less than 0.05 were considered statistically significant. All analyses were performed by SAS software version 9.4 (SAS Institute Inc., Cary, NC, USA).

## Results

### Incidence of macrosomia among study subjects

Demographic characteristics, lifestyle, and environmental exposures of the study population are summarized in Table 1. A total of 105,768 live births were included in the final analysis where 12,335 (11.66%) macrosomic infants were identified. A higher proportion of macrosomia was observed in male fetuses, multiparous, maternal age of 35 years and above, mothers with lower educational level (high school and below), maternal BMI above 24.9 kg/m^2^, and current and previous GDM. Besides, women without anemia and preterm birth more often had macrosomia, while other factors such as paternal smoking, paternal and maternal drinking, and maternal thyroid disease might not contribute to substantial differences in macrosomia prevalence. Due to the very low prevalence rate of maternal smoking (before gestation 0.66% [*n*=693], during gestation 0.17% [*n*=176]), this factor was not included in the analysis.

**Table 1:** Demographic, lifestyle, and clinical characteristics of pregnant mothers

Characteristics	N	Macrosomia (%)	Grade 1 macrosomia (%)	Grade 2 macrosomia (%)	Grade 3 macrosomia (%)
**Overall**	105,768	12,335 (11.66)	10,925 (10.33)	1121 (1.06)	289 (0.27)
**Fetal sex**					
Male	54,382	7730 (14.21)	6811 (12.52)	740 (1.36)	179 (0.33)
Female	51,386	4605 (8.96)	4114 (8.01)	381 (0.74)	110 (0.21)
**Residence**					
Urban	78,232	8793 (11.24)	7785 (9.95)	807 (1.03)	201 (0.26)
Rural	27,504	3541 (12.87)	3139 (11.41)	314 (1.14)	88 (0.32)
**Maternal age group, years**					
20**–**24	11,332	1223 (10.79)	1108 (9.78)	79 (0.70)	36 (0.32)
25**–**29	36,578	3993 (10.92)	3588 (9.81)	323 (0.88)	82 (0.22)
30**–**34	38,649	4627 (11.97)	4068 (10.53)	451 (1.17)	108 (0.28)
≥35	19,209	2492 (12.97)	2161 (11.25)	268 (1.40)	63 (0.33)
**Educational level**					
Secondary school or below	20,610	2760 (13.39)	2435 (11.81)	248 (1.20)	77 (0.37)
High school	22,603	2996 (13.25)	2625 (11.61)	296 (1.31)	75 (0.33)
College/university	56,547	6094 (10.78)	5433 (9.61)	537 (0.95)	124 (0.22)
Postgraduate	5997	484 (8.07)	431 (7.19)	40 (0.67)	13 (0.22)
**Occupational physical activity**					
Light	65,350	7192 (11.01)	6399 (9.79)	638 (0.98)	155 (0.24)
Moderate	28,968	3672 (12.68)	3220 (11.12)	359 (1.24)	93 (0.32)
Active	11,450	1471 (12.85)	1306 (11.41)	124 (1.08)	41 (0.36)
**Pre-pregnancy BMI group, kg/m**^**2**^					
<18.5	10,352	527 (5.09)	493 (4.76)	17 (0.16)	17 (0.16)
18.5–24.9	72,669	7622 (10.49)	6864 (9.45)	602 (0.83)	156 (0.21)
25.0–29.9	18,622	3358 (18.03)	2870 (15.41)	402 (2.16)	86 (0.46)
≥30.0	4125	828 (20.07)	698 (16.92)	100 (2.42)	30 (0.73)
**Parity**					
Nulliparous	51,261	5404 (10.54)	4835 (9.43)	461 (0.90)	108 (0.21)
Multiparous	54,507	6391 (12.72)	6090 (11.17)	660 (1.21)	181 (0.33)
**Current GDM**					
Yes	16,829	2377 (14.12)	2037 (12.10)	269 (1.60)	71 (0.42)
No	88,939	9958 (11.20)	8888 (9.99)	852 (0.96)	218 (0.25)
**Previous GDM**					
Yes	4179	695 (16.63)	571 (13.66)	94 (2.25)	30 (0.72)
No	52,435	6492 (12.38)	5747 (10.96)	587 (1.12)	158 (0.30)
**Maternal drinking before or during pregnancy**					
Yes	2134	252 (11.81)	214 (10.03)	32 (1.50)	6 (0.28)
No	103,634	12,083 (11.66)	10,711 (10.34)	1089 (1.05)	283 (0.27)
**Paternal drinking before pregnancy**					
Yes	34,349	4225 (12.30)	3736 (10.88)	397 (1.16)	92 (0.27)
No	70,423	7987 (11.34)	7083 (10.06)	715 (1.02)	189 (0.27)
**Paternal smoking before pregnancy**					
Yes	40,693	5045 (12.40)	4424 (10.87)	494 (1.21)	127 (0.31)
No	65,075	7290 (11.20)	6501 (9.99)	627 (0.96)	162 (0.25)
**Maternal co-morbidities**					
**Anemia**					
Yes	1934	163 (8.43)	137 (7.08)	20 (1.03)	6 (0.31)
No	103,834	12,172 (11.72)	10,788 (10.39)	1101 (1.06)	283 (0.27)
**Thyroid disease**					
Yes	4054	433 (10.68)	389 (9.60)	32 (0.79)	12 (0.30)
No	101,714	11,902 (11.70)	10,536 (10.36)	1089 (1.07)	277 (0.27)
**Preterm birth**					
Yes	4135	42 (1.02)	26 (0.63)	10 (0.24)	6 (0.15)
No	101,408	12,272 (12.10)	10,882 (10.73)	1108 (1.09)	282 (0.28)

### Stratified analyses

Albeit multiparous mothers (51.53%) had an increased risk of giving birth to a macrosomia infant (OR=1.24, 95% CI 1.19–1.28), the interactive effect between pre-pregnancy BMI and maternal age on macrosomia was statistically significant in both nullipara (*P*_*adjusted*_=0.0265) and multipara (*P*_*adjusted*_=0.0356). We further evaluated the association between macrosomia and pre-pregnancy BMI stratifying by maternal age, parity, and fetal genders (Table 2). A significantly increased risk of macrosomia is in mothers who were overweight or obese in both nullipara and multipara (adjusted *P* values all <0.05). Maternal low BMI (for nullipara: adjusted OR [aOR]= 0.47, 95% CI 0.42–0.53; for multipara, aOR=0.45, 95% CI 0.39–0.52) was inversely associated with macrosomia, and such association was consistent across all maternal ages and in both fetal genders. Specifically, the effect of pre-pregnancy BMI on macrosomia varied with maternal age in both nullipara and multipara. Nulliparous women aged 35 years and above were most benefited from a lower pre-pregnancy BMI against fetal macrosomia (aOR=0.16, 95% CI 0.05–0.49) compared with women of other age groups, whereas multiparous mothers younger than 25 years of age who were low BMI before pregnancy had the lowest risk for macrosomia (aOR=0.17, 95% CI 0.05–0.55). The inverse relationship between low BMI and macrosomia also holds true regarding GDM status in nullipara (non-GDM: aOR= 0.47, 95% CI 0.42–0.53; GDM: aOR= 0.48, 95% CI 0.33–0.70). As for multipara, a lower BMI did not appear to be a protective factor against macrosomia considering GDM status (previous GDM: aOR= 0.81, 95% CI 0.43–1.52; previous and current GDM: aOR= 0.41, 95% CI 0.16–1.04).

**Table 2:** Association between pre-pregnancy BMI and macrosomia according to logistic regression analysis

Variable	Odds ratios (95%CI)			
	BMI < 18.5kg/m^2^	18.5 kg/m^2^ ≤ BMI ≤ 24.9 kg/m^2^	25.0 kg/m^2^ ≤ BMI ≤ 29.9 kg/m^2^	BMI ≥ 30.0 kg/m^2^
**Nullipara**				
Overall	**0.47 (0.42–0.53)**	1.00	**1.93 (1.80–2.08)**	**2.31 (2.03–2.63)**
GDM status				
Non-GDM	**0.47 (0.42–0.53)**	1.00	**1.92 (1.77–2.08)**	**2.17 (1.87–2.53)**
GDM	**0.48 (0.33–0.70)**	1.00	**2.02 (1.71–2.39)**	**2.81 (2.17–3.65)**
Maternal age, years				
20–24	**0.64 (0.52–0.79)**	1.00	**2.22 (1.89–2.61)**	**2.14 (1.60–2.86)**
25–29	**0.44 (0.37–0.53)**	1.00	**1.95 (1.75–2.17)**	**2.40 (1.97–2.92)**
30–34	**0.39 (0.29–0.51)**	1.00	**1.71 (1.48–1.97)**	**2.35 (1.84–3.00)**
≥35	**0.16 (0.05–0.49)**	1.00	**1.98 (1.56–2.52)**	**2.10 (1.33–3.34)**
Fetal sex				
Male	**0.49 (0.42–0.57)**	1.00	**1.81 (1.64–1.99)**	**2.01 (1.68–2.39)**
Female	**0.45 (0.37–0.54)**	1.00	**2.13 (1.91–2.39)**	**2.80 (2.30–3.39)**
**Multipara**				
Overall	**0.45 (0.39–0.52)**	1.00	**1.81 (1.71–1.93)**	**2.00 (1.80–2.23)**
GDM status				
Without GDM	**0.43 (0.36–0.51)**	1.00	**1.76 (1.64–1.89)**	**1.86 (1.63–2.13)**
Current GDM only	**0.49 (0.30–0.79)**	1.00	**1.84 (1.59–2.14)**	**2.04 (1.60–2.59)**
Previous GDM only	0.81 (0.43**–**1.52)	1.00	**2.14 (1.58–2.89)**	**2.43 (1.39–4.24)**
Current and previous GDM	0.41 (0.16**–**1.04)	1.00	**2.22 (1.73–2.86)**	**3.20 (2.18–4.72)**
Maternal age, years				
20–24	**0.17 (0.05–0.55)**	1.00	**1.67 (1.04–2.69)**	1.49 (0.68**–**3.23)
25–29	**0.46 (0.35–0.61)**	1.00	**1.84 (1.60–2.11)**	**2.25 (1.79–2.82)**
30–34	**0.45 (0.36–0.56)**	1.00	**1.96 (1.79–2.14)**	**2.14 (1.82–2.51)**
≥35	**0.54 (0.37–0.77)**	1.00	**1.60 (1.44–1.78)**	**1.68 (1.37–2.06)**
Fetal sex				
Male	**0.44 (0.37–0.53)**	1.00	**1.77 (1.63–1.91)**	**1.82 (1.58–2.10)**
Female	**0.47 (0.36–0.60)**	1.00	**1.87 (1.70–2.06)**	**2.28 (1.92–2.69)**

Maternal age, GDM status, residence, age groups, education level, pre-pregnancy BMI group, alcohol drinking before or during pregnancy, paternal smoking before pregnancy, paternal drinking before pregnancy, occupational physical activity, preterm birth, anemia, and thyroid diseases

### Combined effect of GDM status and parity in different age groups

**Fig. 2:**
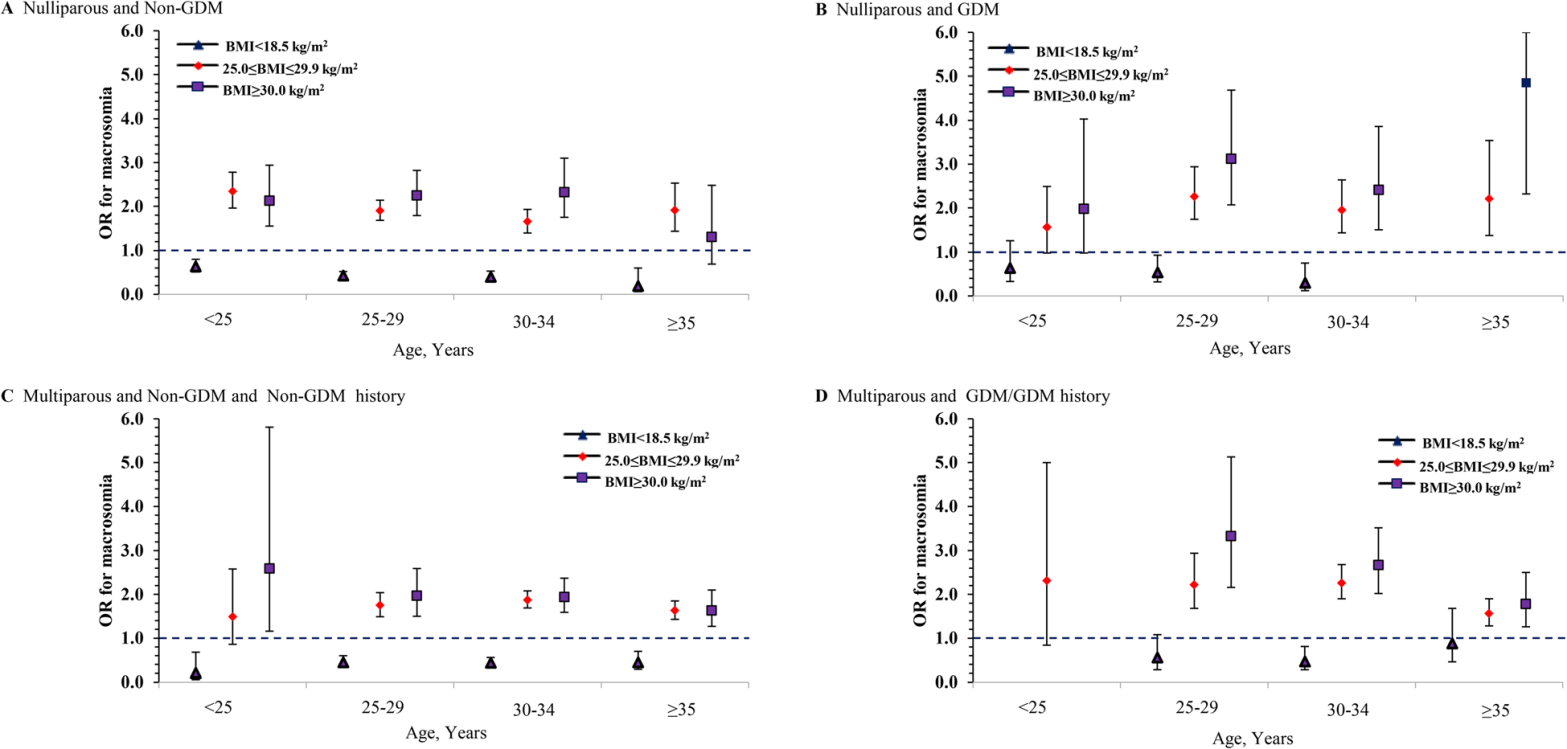
Association between pre-pregnancy BMI and macrosomia stratified by maternal age, parity, and GDM status

To further determine the relationship between pre-pregnancy BMI and fetal macrosomia, other maternal factors including parity, maternal age, and GDM status were jointly evaluated in Fig. 2. In nullipara without GDM, a lower BMI was inversely associated with macrosomia across all age groups (Fig. 2A). In particular, elder mothers were more benefited from a lower BMI (<18.5 kg/m^2^) as the adjusted odds ratios for macrosomia gradually declined from 0.64 (95% CI 0.51–0.80) in the 20-to-24-year-old group to 0.43 (95% CI 0.36–0.52) in the 25-to-29-year-old group to 0.40 (95% CI 0.29–0.53) in the 30-to-34-year-old group and hit the bottom of only 0.19 (95% CI 0.06–0.60) among those aged 35 years and above. A similar pattern could also be observed in nulliparous mothers with GDM, but the magnitude of the effect was less pronounced (Fig. 2B). Among multiparous mothers without GDM (neither current nor previous GDM), low BMI was also a protective factor for macrosomia, but contrary to nulliparous mother, the protection was more significant in the younger age group (20-to-24-year-old group: aOR=0.21, 95% CI 0.06–0.68; 25-to-29-year-old group: aOR=0.45, 95% CI 0.33–0.60; 30-to-34-year-old group: aOR=0.44, 95% CI 0.34–0.56; ≥35-year-old group: aOR=0.45 95% CI 0.29–0.70) (Fig. 2C). In multipara with current and/or previous GDM, the protective effect of low BMI only reached a significant level among those aged 30–34 years (aOR=0.47, 95% CI 0.28–0.81) but not the other age groups (Fig. 2D). Among pregnant women who had current and/or previous GDM, none of nullipara aged 35 years with a BMI below 18.5 kg/m^2^ nor multipara aged 20-to-24 years with a BMI below 18.5 kg/m^2^ or above 30.0 kg/m^2^ gave birth to a macrosomic neonate. Hence, these groups of subjects were not shown in Fig. 2.

## Discussion

In this city-wide population-based cohort study of mother-child pairs, we reported for the first time that the effect of pre-pregnancy BMI on macrosomia varied by maternal age and parity. For nulliparous mothers, the protective effect of a lower pre-pregnancy BMI (especially BMI <18.5 kg/m^2^) against the development of fetal macrosomia was most prominent among those aged 35 years and above, whereas younger multiparous mother was more benefited from a lower BMI. Notably, the association remained statistically significant after stratification and in nulliparous mothers the magnitude of protection related to BMI <18.5 kg/m^2^ increased with conception age, irrespective of GDM status. While among multiparous mother with current and/or previous GDM, the protective effect of a lower pre-pregnancy BMI against macrosomia was only observed in those aged 30–34 years. These findings collectively suggest a differential effect of BMI <18.5 kg/m^2^ on fetal macrosomia concerning maternal age and parity.

Previous findings on the association between pre-pregnancy low BMI and fetal macrosomia were inconclusive. Liu and colleagues systematically reviewed 60 studies involving 1,392,799 pregnant women and also reported an inverse association between pre-pregnancy low BMI and macrosomia [15], whereas a recent cohort study of 2210 women failed to find such association [16]. The discrepancy of findings might be due to a smaller sample size in the latter study. Moreover, previous studies on a similar topic mainly examined the general effect of pre-pregnancy BMI on fetal macrosomia irrespective of maternal age, parity, or GDM status. In the current study, the city-wide population of mother-child pairs allowed us to detect the differential effect based on comprehensive information.

The effect of pre-pregnancy low BMI on macrosomia differed in nullipara and multipara highlight the role of parity in disease pathogenesis. A growing body of evidence has pointed to a positive association between parity and fetal macrosomia [24–27]. A multi-center study conducted in 23 Asian countries documented a significantly increased risk of macrosomia associated with parity (parity 2–4: OR=1.48 [1.41–1.56]; parity ≥5: OR=2.02 [1.76–2.32]) [8]. Structural factors may limit the uterine capacity of nulliparous mothers, but once they become multiparous, their uterine size could increase with accumulated protein contents [28]. Additionally, fetuses of nulliparous mothers were more likely to be exposed to a different maternal immune environment which might restrict their growth in the uterine [29]. Another possible explanation is that multiple parturition is associate with an increased risk of GDM [30] and would aggravate the development of fetal macrosomia. It has been well recognized that GDM plays an important role in the pathogenesis of macrosomia. Approximately 15–45% of pregnant women with GDM develop fetal macrosomia, a 3-fold risk compared to that of non-GDM mothers [31]. Maternal hyperglycemia, the predominant feature of GDM, leads to hyperinsulinemia and increased utilization of glucose in the fetus, resulting in the accumulation of fetal adipose tissue. High concentration of circulating glucose drives the glucose to pass through the placenta, whereas the maternal-derived or exogenously administered insulin cannot cross the placenta. During the second trimester, the fetal pancreas starts to secrete insulin in an autonomous fashion. The combinative effect of hyperinsulinemia and hyperglycemia leads to the accumulation of fat and protein in the fetus, ending up with macrosomia. As multiple parturition is a risk factor for fetal macrosomia, more attention on weight reduction should be paid to tailor the needs for multiparous mothers with a higher BMI.

It is worth noting that although a lower pre-pregnancy BMI was a protective factor for macrosomia across all maternal age groups, such protection was more prominent in elder nulliparous mothers and younger multiparous mothers. The association remained statistically significant after adjusting for the well-known confounders including GDM status, residence, education level, maternal drinking before and/or during pregnancy, paternal drinking and/or smoking before pregnancy, occupational physical activity, preterm birth, anemia, and thyroid diseases, suggesting an independent effect. To the best of our knowledge, this is the first population-based cohort study with a focus on the differential effect of pre-pregnancy BMI and macrosomia. Our findings provide a new insight on modifiable factors for the development of preventative strategies, and further investigations are needed to elucidate the exact mechanisms underlying this phenomenon.

The main strength of the current study is the large sample size covering a city-wide population, allowing us to screen all the pregnant mothers in Qingdao during a certain period without selection bias. The superior statistical power enabled us to detect the differential effect of pre-pregnancy BMI on fetal macrosomia in subpopulations, which may not be achieved by other studies with fewer study subjects. There are indeed several limitations in this study. First, we did not measure the gestational weight gain of the pregnant mothers. Nevertheless, a previous study suggested that pre-pregnancy BMI correlated more with neonatal birth weight than gestational weight gain (pre-pregnancy BMI: adjusted r^2^=0.88, gestational weight gain: r^2^= 0.30 ) [32]. Moreover, 14,629 pregnant women were excluded from the analysis due to missing data on 75 g OGTT, which might bias the results. The proportion of missing data in this study is only 10.48%; therefore, we believe that such limitation would not affect our final conclusion. We neither measured the blood pressure of all pregnant women, hence hindering the evaluation of the association of pregnancy-induced hypertension on macrosomia. Although many potential confounders were adjusted, there might still remain some residual effects related to unknown factors.

## Conclusion

In conclusion, maternal low BMI is inversely associated with macrosomia irrespective of maternal age and parity. The impact of pre-pregnancy BMI on fetal macrosomia differed with maternal age and parity. The protective effect of a lower pre-pregnancy BMI against macrosomia was more significant in elder nulliparous mothers and younger multiparous mothers. Therefore, a maintained optimal body weight campaign designed for women preparing for pregnancy should be tailored for maternal age, parity, and previous GDM history. Further studies are warranted to identify the relevant pathways and underlying mechanisms contributing to the differential effects of pre-pregnancy BMI on macrosomia.

## Data Availability

All data supporting our findings are contained in the paper. The datasets used and analyzed during the current study are available from the corresponding author on reasonable request.

## Abbreviations

aOR: Adjusted odds ratio
BMI: Body mass index
CI: Confidence intervals
GDM: Gestational diabetes mellitus
IADPSG: International Association of Diabetes and Pregnancy Study Group recommendation
OGTT: Oral glucose tolerance test
